# Pulmonary Embolism in COVID-19: Trends from a Single-Center Study Across Ten Pandemic Waves in Romania

**DOI:** 10.3390/microorganisms13071634

**Published:** 2025-07-10

**Authors:** Alexandra Herlo, Adelina Raluca Marinescu, Talida Georgiana Cut, Ruxandra Laza, Adina Maria Marza, Claudia Raluca Balasa Virzob, Cristian Iulian Oancea, Lucian-Flavius Herlo, Ioana-Melinda Luput-Andrica, Voichita Elena Lazureanu

**Affiliations:** 1 Department XIII, Discipline of Infectious Diseases, Victor Babes University of Medicine and Pharmacy Timisoara, E. Murgu Square, Nr. 2, 300041 Timisoara, Romania; alexandra.mocanu@umft.ro (A.H.); laza.ruxandra@umft.ro (R.L.); lazureanu.voichita@umft.ro (V.E.L.); 2 Department of Surgery, Victor Babes University of Medicine and Pharmacy Timisoara, E. Murgu Square, Nr. 2, 300041 Timisoara, Romania; marza.adina@umft.ro; 3 Emergency Department, Emergency Clinical Municipal Hospital, 300079 Timisoara, Romania; 4 Department of Clinic Nursing, Victor Babes University of Medicine and Pharmacy Timisoara, E. Murgu Square, Nr. 2, 300041 Timisoara, Romania; virzob.claudia@umft.ro; 5 Department XIII, Discipline of Pneumology, Victor Babes University of Medicine and Pharmacy Timisoara, E. Murgu Square, Nr. 2, 300041 Timisoara, Romania; oancea@umft.ro; 6 Center for Research and Innovation in Precision Medicine of Respiratory Diseases (CRIPMRD), Victor Babes University of Medicine and Pharmacy Timisoara, E. Murgu Square, Nr. 2, 300041 Timisoara, Romania; 7 Doctoral School, Victor Babes University of Medicine and Pharmacy Timisoara, 300041 Timisoara, Romania; flavius.herlo@umft.ro (L.-F.H.); ioana.luput-andrica@umft.ro (I.-M.L.-A.)

**Keywords:** COVID-19, pulmonary embolism, pandemic waves, thrombosis, inflammatory markers

## Abstract

The COVID-19 pandemic has significantly impacted global health, with pulmonary embolism (PE) emerging as a critical complication due to the hypercoagulable state induced by SARS-CoV-2 infection. Despite advancements in prevention and treatment, PE remains a major cause of morbidity and mortality in COVID-19 patients. This study analyzes the trends, outcomes, and contributing factors of PE across ten pandemic waves in Romania, highlighting the evolving clinical burden and management approaches. This retrospective observational study was conducted on confirmed COVID-19 patients that also developed PE, who were admitted to “Victor Babeș” Hospital and Municipal Emergency Hospital in Timișoara, Romania. Data on demographics, clinical features, inflammatory markers, comorbidities, and treatment were collected from medical records. Statistical analyses, including ANOVA and Kaplan–Meier survival analysis, were conducted to evaluate trends and survival outcomes over time. The study included 166 patients, with a mean age of 67.26 ± 13.57 years. Mortality peaked at 50% in Wave 1, declined to 12% in Wave 7, and increased again to 28% in Wave 10. Intubation rates varied, with a high of 29% in Wave 6 and a low of 12% in Wave 8. Lung involvement was the most severe in Wave 4 (mean 0.54 ± 0.18) but improved in later waves, reaching a mean of 0.24 ± 0.12 in Wave 8. This study highlights the dynamic trends in PE during the COVID-19 pandemic in Romania. Improved clinical management, vaccination, and adaptive healthcare strategies contributed to better outcomes in later waves.

## 1. Introduction

The COVID-19 pandemic has posed unprecedented challenges to global healthcare systems since its emergence in late 2019 [1]. In Romania, as in many other countries, the pandemic evolved through distinct waves characterized by varying viral strains, disease severity, and healthcare burdens [2]. Each wave not only tested the resilience of the medical infrastructure but also revealed unique patterns of complications, such as the increased incidence of PE among patients with severe SARS-CoV-2 infection [3].

Pulmonary embolism has emerged as a critical complication in COVID-19, often associated with the virus’s hypercoagulable state and endothelial damage [4]. In a study conducted by Ng et al., the weighted average incidence of PTE in critically ill COVID-19 patients admitted to the ICU for treatment was 11.1%, even though almost all patients received at least prophylactic doses of anticoagulation therapy [5]. Diagnosing PE in these patients is particularly challenging due to overlapping clinical features with severe COVID-19 pneumonia, including dyspnea, hypoxemia, and chest pain [6].

### 1.1. Epidemiology

Pulmonary embolism has mortality rates ranging from 10% to 30% within the first month following diagnosis. It affects approximately 900,000 individuals annually in the United States alone, underscoring its significant public health impact [7]. In the majority of cases, PE results from deep vein thrombosis (DVT)—blood clots that form in the legs and subsequently travel to the lungs, where they obstruct pulmonary arteries and compromise respiratory function [8].

### 1.2. Pathogenesis

PE is caused by stasis, vascular injury, and hypercoagulability, according to German physician Rudolf Virchow [9]. Beyond postsurgical and trauma-related instances, stasis may cause most venous thrombosis.

**Figure 1:**
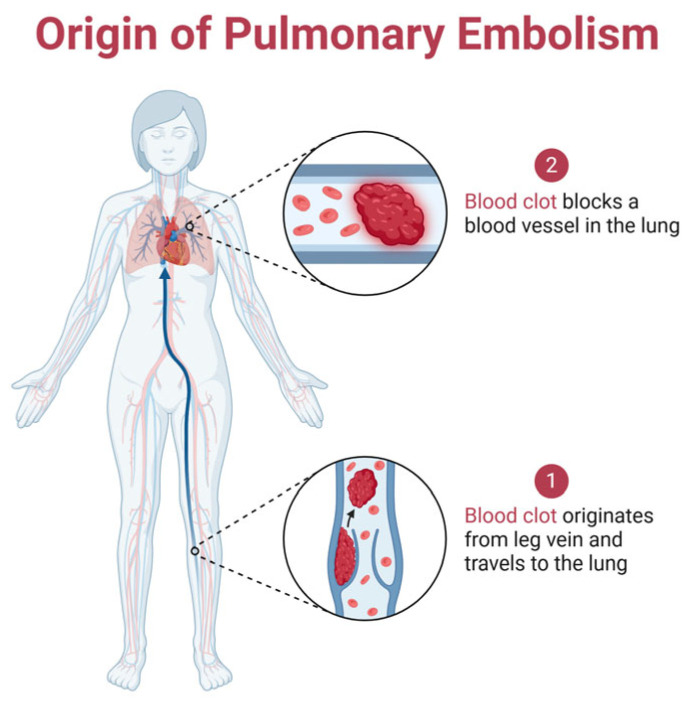
The pathway of pulmonary embolism formation: it begins with a blood clot forming in the leg vein, which then travels through the bloodstream to the lungs, where it blocks a pulmonary artery, disrupting blood flow and potentially leading to life-threatening complications. Created with Biorender [10].

Figure 1 shows the origin of pulmonary embolism and its pathway.

**Figure 2:**
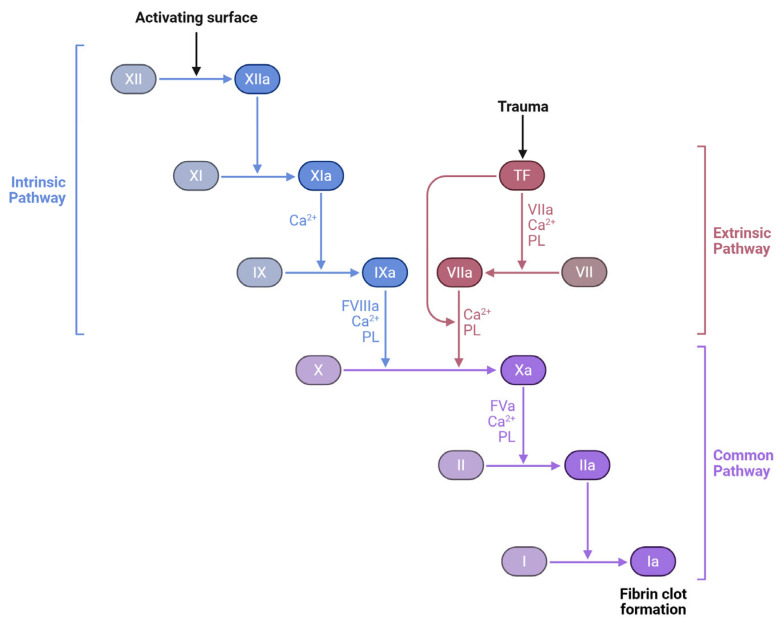
Coagulation cascade: intrinsic, extrinsic, and common pathways. The three major pathways involved in blood coagulation: the intrinsic pathway, initiated by contact activation; the extrinsic pathway, triggered by tissue trauma and tissue factor (TF); and the common pathway, where both converge to produce a fibrin clot. Coagulation factors (e.g., XII, XI, IX, VII, X, II, and I) undergo a sequence of activations, with the aid of calcium ions (Ca^2+^) and phospholipids (PL), culminating in the transformation of fibrinogen (Factor I) into fibrin (Ia), the structural basis of a stable blood clot. Created with Biorender [10].

Thrombosis starts at the valves or sinuses, and venography investigations revealed that contrast media can stay in these locations for 27 min [11]. Autopsy investigations showed that these are the most common thrombosis sites. Venous thrombosis begins with tiny fibrin deposits in low-flow zones. As deposits accumulate, they occlude arteries and cause coagulation cascades as shown in Figure 2. This fibrin nidus can also result from postsurgical or trauma-related endothelial damage [9].

Cartilage oligomeric matrix protein (COMP), also known as thrombospondin-5, is an extracellular matrix protein traditionally associated with cartilage and musculoskeletal tissues. Recent research has identified COMP as an endogenous inhibitor of thrombin, a key enzyme in the coagulation cascade [12].

**Figure 3:**
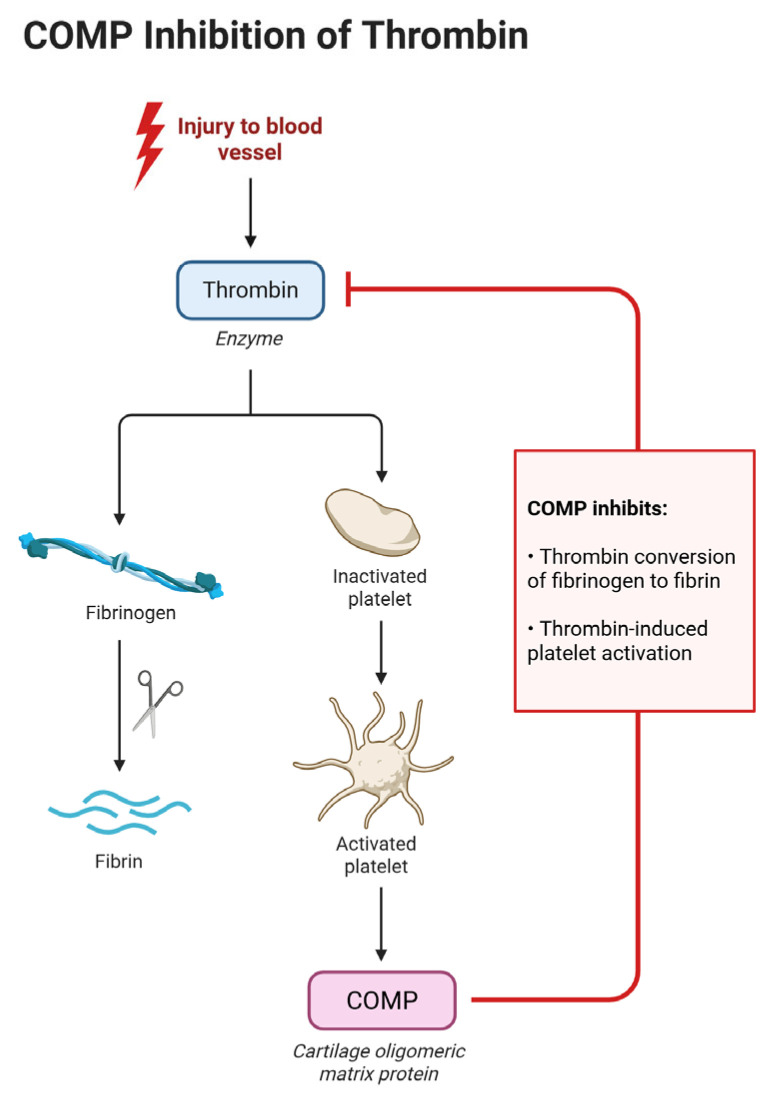
COMP inhibition of thrombin activity: following vascular injury, thrombin is generated and promotes platelet activation and the conversion of fibrinogen to fibrin, leading to clot formation. COMP interferes with this process by binding to thrombin and blocking its ability to activate platelets and convert fibrinogen into fibrin, thus serving as a natural anticoagulant. Created with Biorender [10].

As can be seen in Figure 3, COMP exerts its anticoagulant effects by binding to thrombin’s exosites I and II, thereby inhibiting thrombin-induced platelet aggregation, activation, and the conversion of fibrinogen to fibrin [11]. This interaction serves as a negative feedback mechanism to regulate thrombin activity and limit excessive clot formation.

In the context of pulmonary embolism, the role of COMP is particularly noteworthy. Experimental studies in COMP-deficient mice have demonstrated accelerated thrombus formation and shortened bleeding times, indicating a heightened procoagulant state in the absence of COMP [13].

### 1.3. Pandemic Waves in Romania

Romania’s experience with the pandemic, marked by ten distinct waves from January 2020, offers valuable insights into the evolution of PE cases in COVID-19 patients [14]. Each wave brought its challenges, from the original Wuhan strain in the first wave to the highly transmissible Omicron subvariants in later waves [15]. Factors such as patient demographics, vaccination rates, and comorbidities significantly influenced PE outcomes. For instance, low vaccination coverage during early waves compounded disease severity, while pre-existing conditions like hypertension and diabetes exacerbated thrombotic risks [16].

This retrospective analysis, based on data from the “Victor Babeș” Hospital of Infectious Diseases and Pneumology in Timișoara, Romania, aims to examine the interplay between pandemic waves and the incidence of PE in COVID-19 patients.

### 1.4. Gaps in Knowledge and Disparities in the Management of PE

PE imaging has several sex-dependent differences. Women had more low-yield CT angiograms for PE, exposing them to more radiation and iodinated contrast. Imaging difficulties are also particular to pregnant people. Pregnancy lowers pulmonary CT angiography diagnostic yield due to increased cardiac output and inferior vena cava venous return, which dilutes iodinated contrast.

Race-related treatment discrepancies are linked to socioeconomic inequality. Data reveal that fully insured people utilize direct oral anticoagulation less for VTE treatment and prevention.

Poorer outcomes may be linked to less healthcare access due to longer time to diagnosis, higher severity at presentation, longer time to anticoagulation initiation after PE diagnosis, and less access to advanced interventional and supportive care.

## 2. Materials and Methods

### 2.1. Study Design

The population cohort came from Timisoara’s “Victor Babes” Hospital of Infectious Disease and Pneumology and Municipal Emergency Hospital. The observational retrospective study included patients admitted between March 2020 and December 2024. As part of the retrospective analysis, paper and digital records of patients diagnosed with severe SARS-CoV-2 infection were examined.

During the COVID-19 pandemic, this hospital played a crucial role in managing cases in Bucharest, providing both emergency and specialized care. While specific annual presentation rates are not publicly available, the hospital’s extensive coverage area includes three sectors of Bucharest and six Romanian counties, indicating a significant patient influx and a substantial role in the national healthcare system.

The infectious disease clinic at the “Victor Babes” University of Medicine and Pharmacy, an extension of the Hospital for Infectious Disease and Pneumology in Timisoara, and Municipal Emergency Hospital Timisoara follow local ethical research regulations and technical requirements of the the International Conference on Harmonisation and the Declaration of Helsinki for pharmaceutical registration. On 28 May 2021, the ethics committees of these institutions approved the research with authorization number 9148/3 October 2023 for the “Victor Babes” Hospital for Infectious Disease and Pneumology and 7555/20 December 2023 for Municipal Emergency Hospital Timisoara.

Chart review and data extraction were conducted independently by two trained reviewers using a standardized data collection form. Any discrepancies were resolved through discussion and consensus to ensure accuracy and consistency in data abstraction.

### 2.2. Inclusion and Exclusion Criteria

We examined electronic and physical medical records to determine the prevalence of TEP hospital admissions in SARS-CoV-2 patients. Several journal publications explained sample size selection and its criteria. Palinkas et al.’s criterion sampling method was used to choose participants based on significance criteria [17].

Table 1 shows the inclusion and exclusion criteria for patient selection.

The age cut-off of >51 years was selected to focus the analysis on a population with increased susceptibility to severe COVID-19 and thromboembolic complications, such as pulmonary embolism. This threshold also helped reduce the effect of age-related confounding factors from younger patients who typically present with milder disease and fewer comorbidities.

SARS-CoV-2 infection’s clinical spectrum is classified by the Romanian Ministry of Health, as presented in Table 2 [18].

**Figure 4:**
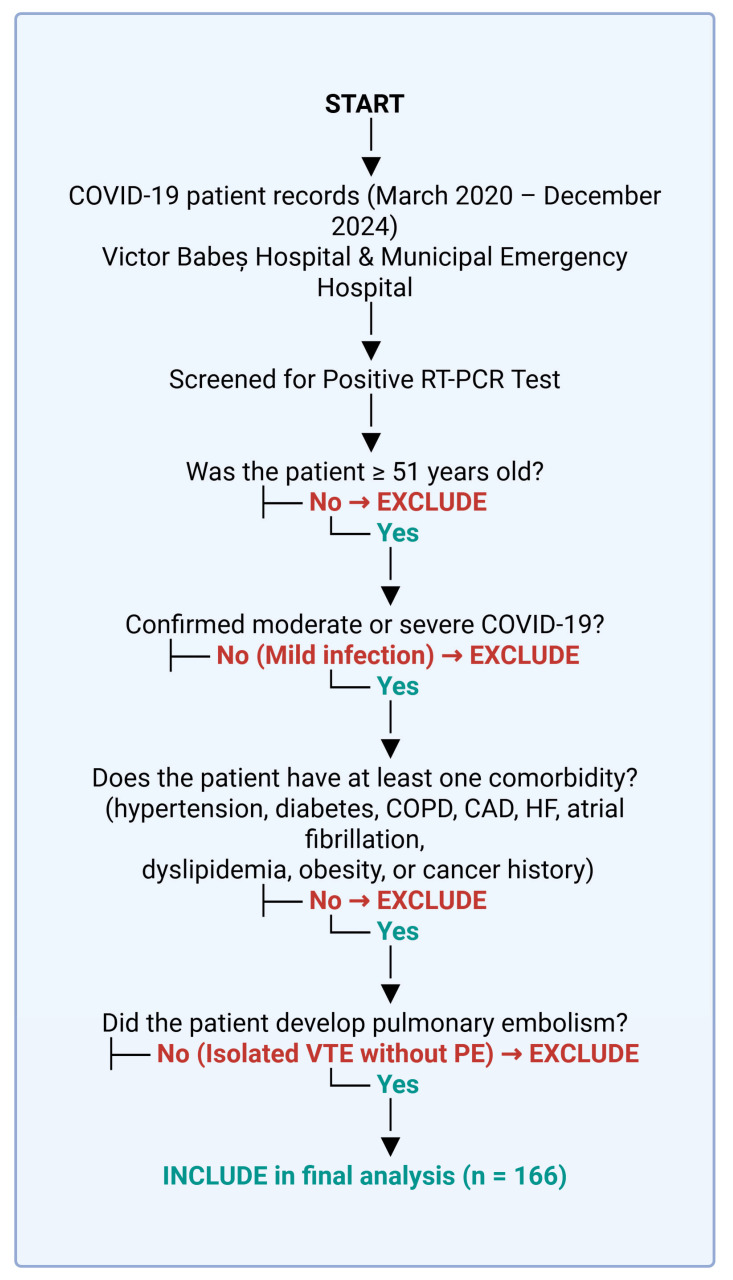
Patient selection process. Created with Biorender [10].

In Figure 4, we present a flowchart for the inclusion and exclusion criteria.

The WHO defines severe infection as SpO_2_ levels below 90%, pneumonia, and respiratory distress, as seen by auxiliary muscle usage and difficulty in finishing phrases [19]. Patients were also categorized by pandemic wave. Romanian Ministry of Health classifications were used to assess pandemic waves [20].

Table 3 shows the pandemic waves in Romania.

### 2.3. Statistical Analysis

The incidence of occurrences was computed for closed cases—discharged, died, or diagnosed with a pulmonary thromboembolic event—with a 95% confidence interval. Using a one-way analysis of variance (ANOVA), inflammatory markers were compared among therapy types and patient outcomes. We examined the F-statistic and *p*-values to determine the statistical significance of group differences in each inflammatory marker. Time-to-event data was analyzed using Kaplan–Meier survival analysis, focusing on patient survival after pulmonary embolism. Survival curves were used to evaluate outcomes, and the log-rank test was used to compare survival distributions. Data analysis was performed using SPSS v. 29.0 [21].

## 3. Results

Table 4 summarizes the demographic, clinical, and laboratory profiles of the patient cohort included in this study. We include an overview of the key characteristics of COVID-19 patients diagnosed with pulmonary embolism, focusing on factors that influence their clinical outcomes.

### 3.1. Demographic and Clinical Characteristics of COVID-19 Patients with Pulmonary Embolism

The demographic and clinical characteristics of COVID-19 patients with pulmonary embolism reveal notable insights. The mean age of the cohort was 67.26 years (±13.57), with a slight male predominance (55.03%). A significant proportion of patients resided in urban areas (63.08%), and obesity was prevalent in 41.89% of cases.

Laboratory findings further highlight the severity of systemic involvement. The mean white blood cell count was elevated (10,893.41 ± 11,593.79), driven primarily by neutrophilia (9298.14 ± 10,869.27), while lymphocyte counts were notably low (885.68 ± 393.62), indicative of a pro-inflammatory state. Markers of inflammation and coagulation were significantly raised, with mean D-dimer levels reaching 4265.20 ng/mL (±3480.29), ferritin levels reaching 1114.47 ng/mL (±921.16), and fibrinogen levels reaching 5.54 g/L (±1.53). C-reactive protein (CRP) and interleukin-6 (IL-6) were also elevated, underscoring the hyperinflammatory milieu. Furthermore, markers of organ function such as lactate dehydrogenase (LDH) at 592.41 ± 270.02 U/L and slightly elevated creatinine (1.04 ± 0.46 mg/dL) suggested multi-organ stress. These characteristics underline the critical nature of pulmonary embolism in COVID-19 patients and the complexity of their clinical management.

### 3.2. Comorbidities and Their Impact on Disease Outcomes

Hypertension was the most common comorbidity, affecting 78.9% of patients, followed by diabetes in 30.72% and pulmonary hypertension in 27.1%. Other notable conditions included COPD (8.43%), cancer (14.45%), and chronic kidney disease (CKD) (13.25%). Functional cardiac capacity, assessed via NYHA classification, showed most patients in NYHA classes 2 (30.13%) and 3 (21.08%), with few in the extremes of NYHA 1 (17.46%) and NYHA 4 (0.6%). Atrial fibrillation (10.84%) and venous insufficiency (12.65%) were other observed cardiac conditions.

### 3.3. Therapeutic Approaches, Vaccination Status, and Outcomes in Patients with COVID-19 and Pulmonary Embolism

Therapeutic approaches in COVID-19 patients with pulmonary embolism focused on anticoagulation and antiviral therapies alongside supportive measures. Enoxaparin was the most frequently administered anticoagulant, used in 36.74% of patients, followed by Nadroparin (37.95%) and sodium heparin (16.86%). Thrombolysis with actilyse was employed in 6.02% of cases, reflecting its use in severe presentations. Antiviral treatment included Remdesivir in 81.32% of patients and Favipiravir in 16.86%. Therapies such as Dexamethasone (59.04%), Anakinra (35.54%), and Tocilizumab (10.84%) addressed hyperinflammatory states. Vaccination against COVID-19 was recorded in 28.31% of patients, emphasizing the low vaccination coverage within the cohort. Despite aggressive treatment strategies, outcomes were severe, with 24.69% mortality, 18.67% patients requiring intubation, and an equal proportion managed with CPAP.

### 3.4. Statistical Analysis

The one-way analysis of variance (ANOVA) assesses whether statistically significant differences exist among the means of independent groups [15]. For this research, we selected inflammatory markers and anticoagulant therapy as independent groups, and they are organized in Table 5.

The F-value for CRP is 1.077, and the *p*-value is 0.301, signifying no statistically significant difference across the groups. Correspondingly, ESR showed an F-value of 1.506 and a *p*-value of 0.222, which is likewise not statistically significant. The pattern continues for Fibrinogen and Ferritin with F-values of 1.333 and 1.619, respectively, and *p*-values of 0.250 and 0.205, indicating no significant difference among the groups. D-dimers showed the lowest F-value of 0.017 and the highest *p*-value of 0.897, and ultimately, IL-6 had an F-value of 0.260 and a *p*-value of 0.611.

Thus, none of the inflammatory markers included in Table 2 displayed statistically significant differences between the groups, and their levels were comparably consistent among each other, possibly suggesting that these inflammatory markers may not have been significantly affected by the anticoagulant therapy.

Table 6 presents the results of a COX regression analysis that we performed to investigate the relationship between the survival time of the patients and other variables, such as drug therapy and comorbidities. In the context of our study on pulmonary embolism and COVID-19 patients, the Cox regression model helped us to assess the impact of these variables on the survival possibilities of the patients.

Of the total 166 cases included in the study, 41 cases (24.7%) experienced the event of interest (death in our case) during the study period. A total of 122 cases (73.5%) were censored, meaning these patients survived by the end of the study period. Only three cases (1.8%) were dropped because they were censored before any events occurred within their respective strata, which is a minimal loss of data and does not significantly affect the overall analysis. The final sample size for the analysis was 166 cases (98.2%), which is substantial and gives confidence to the statistical power of the analysis.

**Figure 5:**
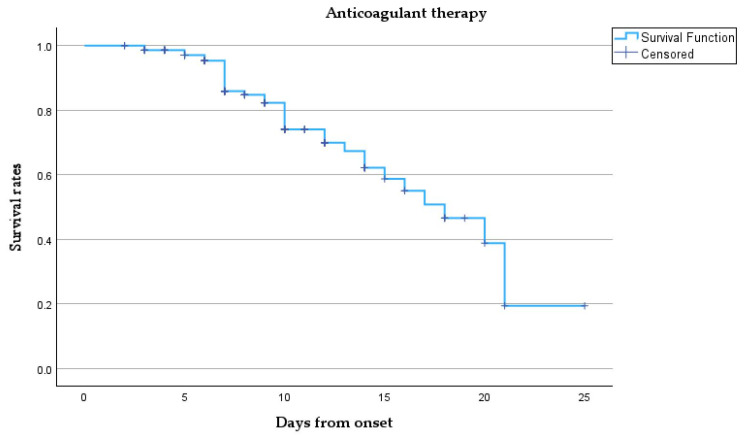
Kaplan–Meier survival curve.

Figure 5 presents a Kaplan–Meier survival analysis.

This survival curve describes the survival probability over time for our cohort treated with anticoagulant therapy. Initially, the survival probability is 1.0 (100%) at the onset, meaning all patients are alive. However, as time progresses, the survival rate gradually declines. Despite the censored points, the overall trend shows a steady reduction in survival probability as more patients died. Notably, between days 10 and 20, the survival rate declines significantly, and by day 25, the probability of survival approaches zero, indicated by the sharp decline towards the end of the curve, meaning that most patients have either died or left the study.

**Figure 6:**
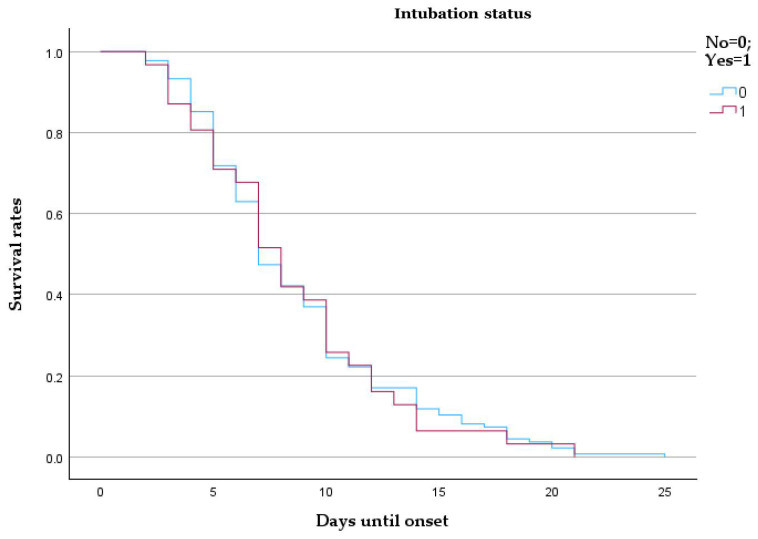
Kaplan–Meier survival analysis comparing intubated and non-intubated patients with pulmonary embolism.

The survival probability curves show minimal difference between the two groups over the first 25 days from onset. Patients requiring intubation (coded as 1) exhibited a slightly lower survival trend than those who were not intubated (coded as 0), though the curves overlap substantially, suggesting limited prognostic separation based on intubation status alone. The mean and median survival times were similar between groups (mean: 8.70 vs. 8.48 days; median: 7 vs. 8 days), with overlapping 95% confidence intervals, as shown by Table 7 and Figure 6. 

**Figure 7:**
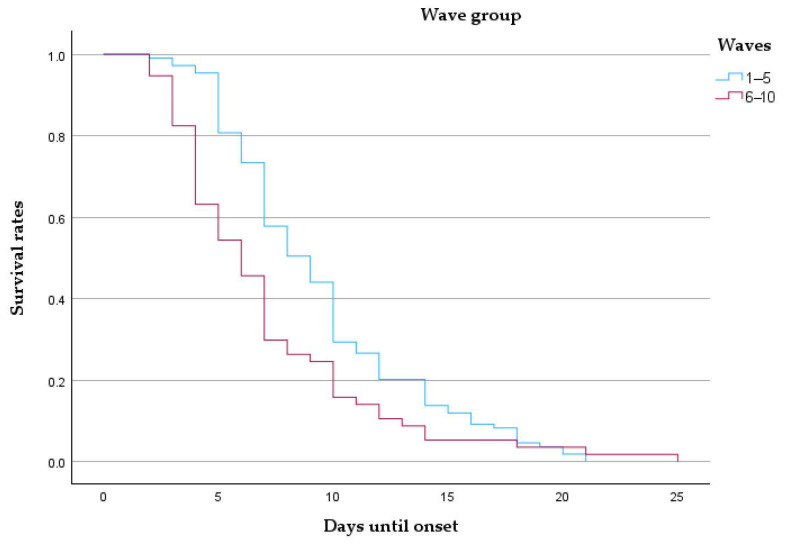
Kaplan–Meier survival curve comparing patients from early (waves 1–5) and late (waves 6–10) pandemic periods.

Patients admitted during the early waves demonstrated notably higher survival probabilities throughout the 25-day follow-up compared to those from later waves. The curve for Waves 6–10 descends more steeply, indicating accelerated mortality. The median survival was 9 days for early waves and 6 days for late waves, with mean survival estimates of 9.48 and 7.09 days, respectively (Table 8 and Figure 7). 

**Figure 8:**
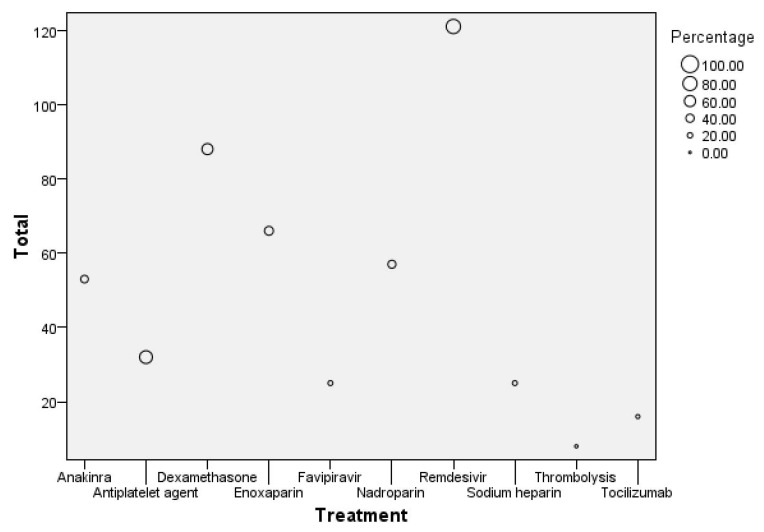
Bubble plot of treatment distribution and patient count.

The bubble plot illustrated in Figure 8 shows the distribution of different treatments administered to patients, with the total number of patients on the y-axis and specific treatments on the x-axis.

The size of each bubble represents the percentage of patients receiving that treatment. The largest bubble is for Remdesivir, with 121 patients receiving this treatment, accounting for the highest percentage in the dataset. Enoxaparin follows with 66 patients, reflecting a significant proportion as well. Other treatments like Dexamethasone and Anakinra also have notable patient counts of around 88 and 53, respectively. In contrast, treatments like Tocilizumab and Thrombolysis have much smaller bubbles, indicating that fewer patients (around 16 for Tocilizumab and 8 for Thrombolysis) received these treatments, representing the lowest percentages in the group.

The drug administration patterns observed in this cohort are reported descriptively. No statistical comparisons were performed to evaluate treatment efficacy, and no causal inferences should be drawn from these data. The findings are presented to illustrate the therapeutic landscape within the study population.

Table 9 shows a comparison of Wells, Pessi, and IMPRUVE-VTE scores across the ten waves. The Wells score predicts deep vein thrombosis and pulmonary embolism in patients. This helps determine whether D-dimer or imaging tests are needed. The PESI (Pulmonary Embolism Severity Index) and simplified PESI (sPESI) are risk assessment tools for patients with acute pulmonary embolism to predict 30-day mortality. The IMPRUVE-VTE (Investigating Modern Prevention and Treatment Strategies in Venous Thromboembolism) study is a clinical trial evaluating new treatment strategies for venous thromboembolism (VTE).

The table reveals distinct patterns that reflect the evolving clinical severity and complexity of patients with pulmonary embolism. Early waves, such as Wave 1 (n = 2), showed lower Wells (mean 0.75 ± 0.75), Pessi (mean 2 ± 1), and IMPRUVE-VTE scores (mean 3 ± 1), indicating a relatively lower risk profile in a small patient group. As the pandemic progressed, these scores increased, with Wave 2 (n = 26) showing a Wells score of 1.3 ± 1.33 and a Pessi score of 3.53 ± 1.24. These metrics highlight a moderate risk elevation as the patient cohort expanded. By Wave 5 (n = 26), the Pessi score rose to 4.07 ± 0.87, and the IMPRUVE-VTE score increased to 4.88 ± 1.28, suggesting a trend toward higher risk and disease burden.

Later waves, particularly Waves 6 through 10, exhibited substantial variability and higher scores, reflecting an increasingly severe patient profile. For instance, Wave 7 (n = 15) demonstrated a sharp rise in the Wells score (mean 2.53 ± 2.08), coinciding with a high Pessi score of 4.25 ± 1.08. Similarly, Wave 9 (n = 9) reported the highest Pessi score (mean 4.88 ± 0.31) and a notable IMPRUVE-VTE score of 5.66 ± 1.15, indicating a critical level of thrombotic risk. By Wave 10 (n = 7), Wells scores stabilized at 1.42 ± 0.82, but IMPRUVE-VTE scores remained high (5.71 ± 0.45). This progression underscores the increasing severity and complexity of cases over time, emphasizing the need for targeted interventions in later waves to mitigate elevated risks.

Table 10 shows the Kendall’s tau-b correlation coefficient between various paired comparisons of the means of the Wells, Pessi, and IMPRUVE-VTE scores, as well as their correlation with the study waves.

The correlation between the Wells and Pessi scores shows a moderate positive association (tau-b = 0.619), but with a *p*-value of 0.051, it narrowly misses statistical significance. The confidence interval (−0.025 to 0.900) crossing zero further supports the conclusion that the correlation is not significant. Thus, one is not influenced by the other when it comes to the waves of the COVID-19 pandemic. Similarly, the correlation between Wells and IMPRUVE-VTE (tau-b = 0.524) scores and the correlation between Wells scores and the waves (tau-b = 0.524) also show moderate positive relationships, but both lack statistical significance, with *p*-values of 0.099 and confidence intervals crossing zero.

In contrast, the Pessi and IMPRUVE-VTE scores exhibit a strong positive correlation (tau-b = 0.905) with a *p*-value of 0.004, indicating statistical significance. The 95% confidence interval (0.635 to 0.978) does not cross zero, confirming a significant and strong relationship between these two scoring systems. Additionally, both the Pessi score (tau-b = 0.905, *p* = 0.004) and the IMPRUVE-VTE score (tau-b = 0.810, *p* = 0.011) show strong, statistically significant correlations with the pandemic waves, with confidence intervals that further support the strength and significance of these associations.

**Figure 9:**
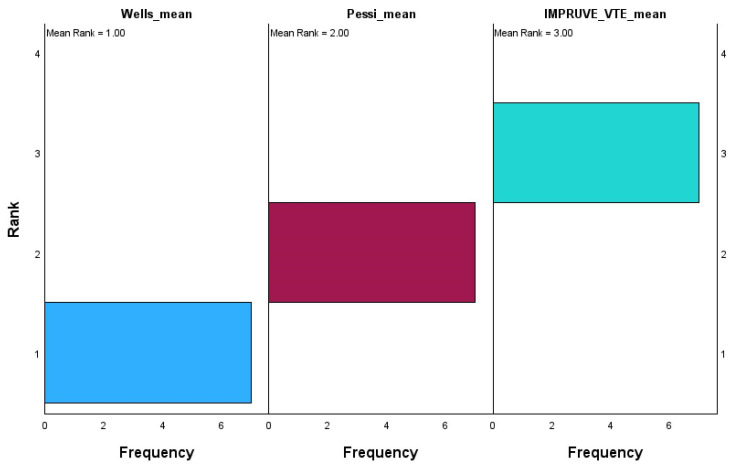
Related samples: Friedman’s two-way analysis of variance by ranks.

The chart in Figure 9 displays the ranks for each scoring system, with their corresponding frequencies on the x-axis and rank values on the y-axis.

The Wells score has the lowest mean rank of 1.00, with most values concentrated at rank 1, indicating that it consistently scores the lowest relative to the other systems. On the other hand, the Pessi score holds a mean rank of 2.00, with its values mainly being centered around rank 2, showing that it occupies an intermediate rank between Wells and IMPRUVE-VTE. The IMPRUVE VTE score has the highest mean rank of 3.00, with most values being concentrated at rank 3, suggesting that it consistently scores higher compared to the Wells and Pessi systems. This indicates that in this dataset, the IMPRUVE-VTE score is relatively higher than both the Wells and Pessi scores, while the Wells score tends to be the lowest. This difference in ranking could reflect variations in how these scoring systems assess patients, with IMPRUVE-VTE potentially being more sensitive or leading to higher values in our cohort.

Table 11 provides a detailed comparison of deaths, intubation rates, and lung involvement imaging features across pandemic waves.

In Wave 1, the sample size was minimal (n = 1), with 50% mortality and intubation rates, alongside limited lung involvement (mean 0.22 ± 0.02). Waves 2 and 3 saw increases in sample size (n = 7 and n = 8, respectively), with mortality rates stabilizing at 27% and 24% and lung involvement imaging features showing a mean of 0.45 in both waves, though with slightly greater variability in Wave 3 (SD 0.24). Mortality remained steady in Waves 4 and 5 at 27% and 23%, respectively, with intubation rates showing minor fluctuations (9% and 15%). Lung involvement imaging peaked at 0.54 ± 0.18 in Wave 4 before declining to 0.35 ± 0.2 in Wave 5. By Wave 6, mortality rose to 35%, accompanied by the highest intubation rate (29%) since the pandemic’s early phases. However, lung involvement imaging features showed only a modest increase (mean 0.31 ± 0.19). Subsequent waves showed reduced mortality and intubation rates, with Wave 7 reporting the lowest mortality (12%) but a notable increase in intubation (25%). Lung involvement imaging features showed a steady decline, reaching a mean of 0.24 ± 0.12 in Wave 8, coinciding with zero mortality. By Wave 10, mortality and intubation rates were both at 28%, while lung involvement reached its highest variability (mean 0.31 ± 0.31).

Although SARS-CoV-2 variant-specific classification would have offered enhanced precision in assessing disease behavior and outcomes, our database did not include variant sequencing data as the standard RT-PCR testing carried out during clinical care did not identify specific viral lineages. Therefore, stratification by pandemic wave was the most consistent and reliable framework available for temporal classification. However, we acknowledge that certain waves contained relatively few events (e.g., deaths in Wave 1, Wave 7, and Wave 10), which may limit the robustness of subgroup comparisons. These limitations were considered when interpreting wave-specific findings.

**Figure 10:**
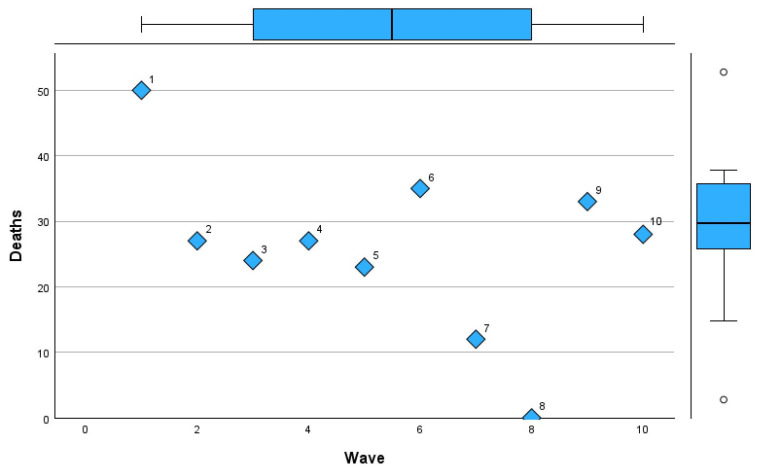
Mortality trends across COVID-19 pandemic waves in Romania.

**Figure 11:**
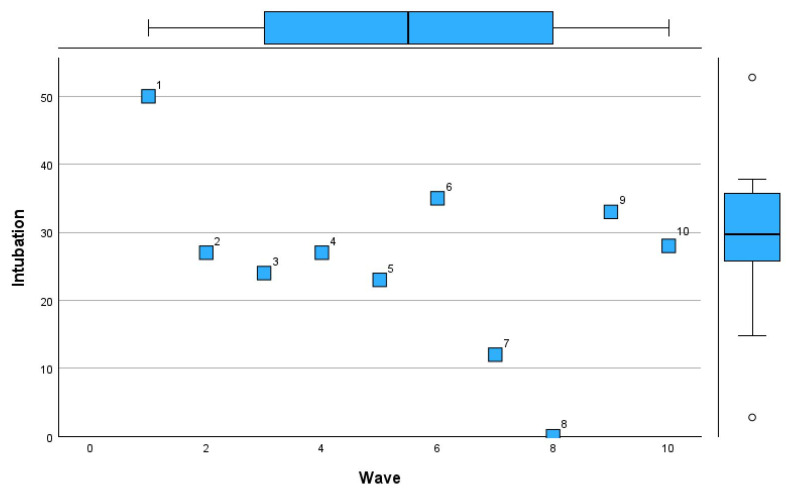
Intubation rate trends across COVID-19 pandemic waves in Romania.

**Figure 12:**
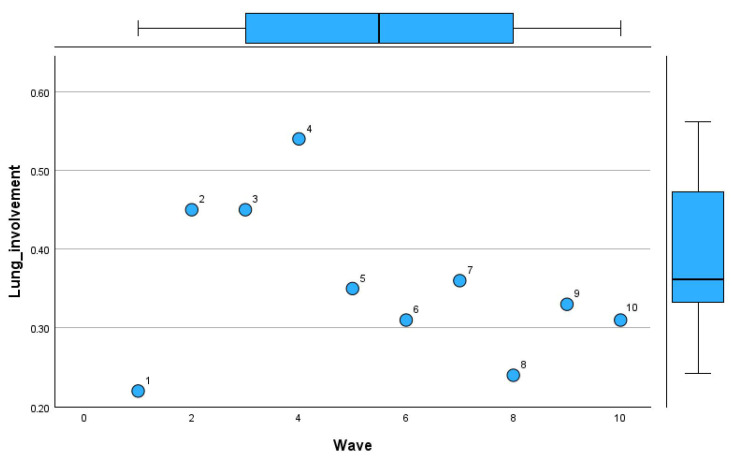
Lung involvement patterns by imaging across COVID-19 pandemic waves in Romania.

Figure 10, Figure 11 and Figure 12 visually represent the trends in deaths, intubation rates, and lung involvement imaging features across different waves of the COVID-19 pandemic. These figures illustrate the evolving clinical burden of pulmonary complications during the pandemic. Figure 10 highlights the variability in death rates across waves.

Wave 1 experienced the highest mortality (50%), reflecting the initial challenges of managing the pandemic. Subsequent waves, particularly Waves 2 to 5, stabilized around a mortality rate of 23–27%, with a notable increase during Wave 6 (35%). Waves 7 to 10 saw reduced mortality, with the lowest rate of 12% in Wave 7 and a slight rebound in Waves 9 and 10 (33% and 28%, respectively).

Figure 11 demonstrates trends in intubation rates.

The initial waves had fluctuating rates, with a peak during Wave 6 (29%). Intubation was least frequent in Wave 8 (12%), coinciding with a zero mortality rate, indicating potentially milder disease during that period. Wave 10 showed a rise in intubation rates (28%), aligning with increased disease severity in later stages of the pandemic.

Figure 12 tracks lung involvement through imaging features.

The mean lung involvement increased from Wave 1 (0.22) to its highest value in Wave 4 (0.54), reflecting severe pulmonary effects during this period. Waves 5 to 8 exhibited a downward trend in lung involvement (mean ~0.31–0.36), suggesting improvements in disease management. However, Wave 10 recorded greater variability, with lung involvement reaching the highest standard deviation (0.31).

Table 12 presents the percentages of pulmonary involvement distributed across waves.

Wave 1 recorded a low number of patents with pulmonary involvement. By Wave 2, the number of patients with pulmonary involvement increased, with 42.3% (11) in the >50% category and 38.4% (10) in the 20–50% category. Wave 3 continued that trend, with 39.3% (13) in the >50% category and 42.4% (14) in the 20–50% category. In Wave 4, the >50% category peaked at its highest percentage, with 59% (13), and only 4.5% (1) showed such a low involvement, defined as being within the 0–20% category.

In subsequent waves, the distribution shifted. By Wave 6, the biggest proportion of patients (47% (8)) had minimal involvement (0–20%), and this persisted through Wave 7 when 46.6% (7) had minimal involvement and only 20% (3) had >50% involvement. Waves 9 and 10 are notable for the dominance of the 0–20% category, with 100% (9) in Wave 9 and 87.5% (7) in Wave 10. This suggests that, in later waves, a marked decrease in severe pulmonary involvement contrasted with earlier waves when >50% involvement was more common.

**Figure 13:**
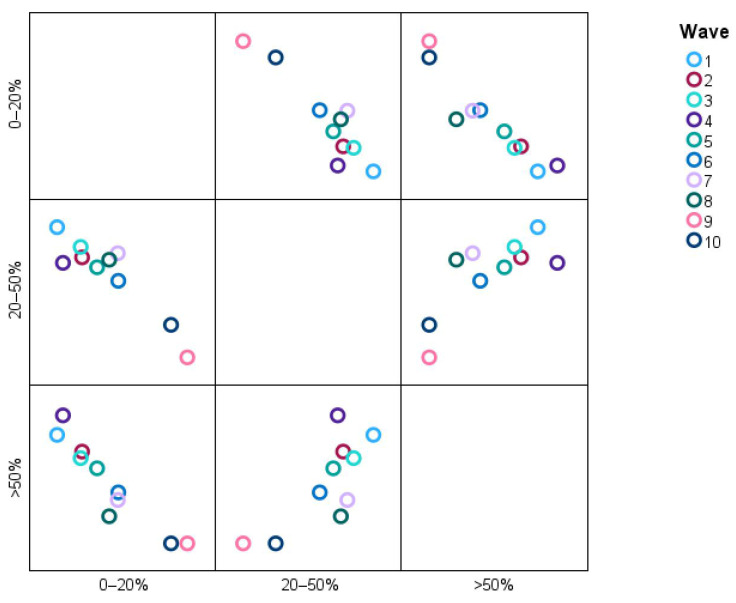
Distribution of pulmonary involvement categories across different waves.

The scatter plot in Figure 13 shows the distribution of categories of pulmonary involvement (0–20%, 20–50%, and >50%) across Waves 1–10.

The data points reveal, for instance, that a greater proportion of patients in Waves 2 and 3 are classified as having >50% pulmonary involvement, whereas the situation in Waves 9 and 10 overwhelmingly describes patients falling into the 0–20% category, meaning that severe pulmonary involvements have dissipated over time. The 20–50% category is uniformly interwoven across all waves, denoting stability or some proportionality with moderate pulmonary involvement. It is an image that quite readily suggests high (>50%) pulmonary involvement decreasing as the condition under study advances.

## 4. Discussion

Prior investigations indicated that COVID-19-infected individuals with pre-existing cardiovascular illness and/or cardiovascular risk factors exhibit a poorer prognosis and are more likely to require admission to the intensive care unit and ventilatory support [20].

These findings may possess significant significance for clinicians. Initially, pulmonary thromboembolism is a likely clinical condition in severe COVID-19 cases, and healthcare professionals should regard all COVID-19 patients as susceptible to venous thromboembolism, particularly when there is delayed hospitalization following symptom onset, a high-risk serum biomarker profile, and echocardiographic indications of right ventricular dysfunction and pulmonary hypertension [21,22]. Timely identification of pulmonary embolism risk factors enables clinicians to initiate immediate, full-dose anticoagulation medication [23]. Secondly, because of the elevated mortality risk, COVID-19 patients with pulmonary embolism should be meticulously monitored throughout hospitalization and, when feasible, admitted to an intensive care unit [24,25,26].

The correlation between COVID-19 and hypercoagulopathy has been documented in the literature [27,28,29,30]. The hypercoagulable state correlates with sickness severity since both the incidence and coagulation abnormalities are more pronounced in severe cases [31,32]. Moreover, the existence of coagulation abnormalities, including higher D-dimer levels, has been associated with both mortality and the requirement for mechanical ventilation [33,34,35].

Hypoxia and inflammation affect valve-expressed antithrombotic proteins such as thrombomodulin and endothelial protein C receptor (EPCR). Hypoxia and high hematocrit levels from valvular sinus stasis create a hypercoagulable environment. These circumstances, particularly acute inflammation, downregulate these proteins and increase thrombus formation [7]. Hypoxia can also upregulate endothelial procoagulants, including tissue factor and P-selectin, which recruit leukocytes or monocyte-derived leukocyte microparticles containing tissue factor. Tissue factor initiates coagulation and is necessary for thrombosis with P-selectin [4]. Without sufficient flow, fibrin deposits activate clotting factors locally, consuming blood coagulation inhibitors without new inhibitors. EPCR and thrombin bound to thrombomodulin activate the protein C pathway, which inactivates cofactors Va and VIIIa [6]. A tissue factor inhibitor inhibits coagulation initiated by tissue factor. Heparin-like proteoglycans promote antithrombin, which blocks thrombin. The venous thrombus is formed by fibrin, red blood cells, and platelets during the coagulation cascade. The red-cell-rich fibrin clot parallel to the endothelium and the platelet-rich white thrombus separate red thrombus regions in the venous clot. Genetic variations that increase coagulation factor VIII, von Willebrand factor, factor VII, and prothrombin levels increase thrombus formation risk [12]. Clinically severe venous thrombosis requires at least two of Virchow’s triad.

The exceptionally high mortality rate of 50% observed during Wave 1 likely reflects the profound strain on healthcare resources during the initial phase of the pandemic. At that time, standardized management protocols were lacking, diagnostic and therapeutic tools were limited, and hospitals faced overwhelming patient volumes, all of which may have contributed to adverse outcomes.

This study provides critical insights into the trends and clinical outcomes of PE in COVID-19 patients during ten pandemic waves in Romania. Mortality, intubation rates, and lung involvement varied significantly across the waves, reflecting the evolving burden of the disease. Mortality peaked at 50% during Wave 1 and was substantially lower in later waves, with a notable decline to 12% in Wave 7. However, higher variability in clinical outcomes during later waves (e.g., mortality at 28% in Wave 10) suggests the impact of viral variants, patient demographics, and healthcare adaptations over time.

Our findings are associated with other studies highlighting the high incidence of thromboembolic complications in COVID-19 patients. For instance, Lodigiani et al. [36] reported a 27.6% prevalence of venous thromboembolism in critically ill COVID-19 patients, emphasizing the hypercoagulable state induced by SARS-CoV-2. Similarly, Zhang et al. [37] observed elevated D-dimer levels and fibrinogen in severe cases, consistent with our cohort’s findings of significantly raised markers of inflammation and coagulation.

Interestingly, vaccination status, which was low in early waves (28.18%), played a role in modulating disease severity. This aligns with data from the RECOVERY Collaborative Group [38], which demonstrated reduced PE incidence and mortality among vaccinated individuals. In our study, improvements in outcomes during later waves coincided with the rollout of vaccination programs and evolving therapeutic approaches, such as the increased use of immunomodulatory therapies like Dexamethasone and Anakinra. Additionally, the pattern of lung involvement observed in our study mirrors the findings by Klok et al. [39], who reported greater pulmonary complications in early pandemic stages. The declining trend in lung involvement imaging features across later waves likely reflects advancements in early detection and treatment strategies [40,41,42].

We emphasize that treatment-related findings in our study are observational and exploratory in nature. Due to the retrospective design and lack of treatment randomization, any apparent differences in outcomes associated with specific therapies must be interpreted with caution. The data reflect clinical decisions made during evolving pandemic conditions and are intended to generate hypotheses rather than establish causality.

These findings point out the dynamic nature of the pandemic and the importance of adapting clinical practices to emerging challenges. Further studies are needed to explore the long-term impact of PE on COVID-19 survivors and the role of evolving therapeutic approaches in mitigating outcomes.

## 5. Strengths and Limitations

This study provides valuable insights into the trends and outcomes of pulmonary embolism (PE) in COVID-19 patients across ten pandemic waves in Romania. A major strength is the comprehensive dataset from “Victor Babeș” Hospital, including detailed demographic, clinical, and laboratory data. This allowed for a robust analysis of variations in PE incidence, mortality, and lung involvement over time. By contextualizing findings within the progression of pandemic waves, the study offers a dynamic view of how evolving viral variants, vaccination rates, and treatment protocols impacted patient outcomes. Furthermore, the study’s focus on a Romanian cohort contributes to understanding COVID-19’s regional effects, complementing global research.

However, there are limitations to consider. As a retrospective analysis, this study relied on existing medical records, which may be subject to data completeness and accuracy issues. The relatively small sample size in certain waves, such as Wave 1 (n = 1) and Wave 8 (n = 0 for deaths), limits the general applicability of results for these periods. Additionally, the absence of long-term follow-up data restricts conclusions on the chronic effects of PE in COVID-19 survivors.

A further limitation of this study is that formal inter-rater reliability testing was not performed during the chart review process. Although the data were reviewed by two independent investigators and a consensus was used to resolve discrepancies, the absence of quantified agreement metrics may introduce the potential for subjective bias.

Another limitation of this study is the absence of systematically recorded echocardiographic data, including left ventricular ejection fraction (LVEF) and detailed heart failure phenotypes. These parameters were not included in the primary database at the time of data collection and, as such, retrospective extraction was not feasible. Consequently, we were unable to classify patients according to heart failure subtypes (e.g., HFrEF and HFpEF), which may have provided additional insights into the cardiopulmonary interplay in COVID-19-associated pulmonary embolism.

Lastly, while comparisons with global studies are valuable, differences in healthcare systems and patient management approaches may influence direct applicability to other populations.

## 6. Conclusions

This study highlights the dynamic trends in PE among COVID-19 patients across ten pandemic waves in Romania. Mortality rates demonstrated significant variation (especially in Wave 1, where the sample size was minimal (n = 2)). During Wave 6, the mortality rate peaked at 35% and declined to 12% in Wave 7, reflecting advancements in clinical management and the introduction of vaccination programs.

However, mortality was 28% in Wave 10, highlighting the persistent challenges of new viral variants and patient comorbidities.

Intubation rates also fluctuated, with the highest rate of 29% being observed in Wave 6. Improvements were noted in later waves, such as Wave 8, where intubation dropped to 12%. These variations emphasize the evolving burden on healthcare systems and the impact of timely interventions. Lung involvement severity, measured by imaging features, peaked during Wave 4 (mean 0.54 ± 0.18) and progressively declined in subsequent waves, reaching a low of 0.24 ± 0.12 in Wave 8.

The study’s findings reveal that vaccination rates, which were only 28.18% in early waves, played a crucial role in mitigating severe outcomes. Moreover, elevated inflammatory markers such as D-dimers highlighted the pro-thrombotic state of severe COVID-19 cases, consistent across waves.

## Figures and Tables

**Table 1:** Inclusion and exclusion criteria.

Inclusion Criteria	Exclusion Criteria
Age ≥ 51 years	Age < 50 years
Confirmed diagnosis of moderate or severe COVID-19 determined by RT-PCR testing	Mild COVID-19 infection
Acute SARS-CoV-2 infection associated with at least one of the following comorbidities: • Hypertension • Diabetes • Chronic Obstructive Pulmonary Disease (COPD) • Coronary Artery Disease (CAD) • Heart Failure • Atrial Fibrillation • Dyslipidemia • Obesity • Cancer History	Patients with isolated venous thromboembolism (VTE) not evolving into PE
Presence of pulmonary embolism	

**Table 2:** Clinical classification of SARS-CoV-2 infection severity.

Parameter	Moderate Infection	Severe Infection
**SpO_2_ (oxygen saturation)**	≥92%	<90%
**Pulmonary involvement (imaging)**	<50% of lung surface	>50% of lung surface
**CRP (C-reactive protein)**	>50 mg/dL	>100 mg/dL
**Ferritin levels**	2–3 × normal values	3–4 × normal values
**D-dimers**	3 × normal (normal < 500 ng/mL or <0.5 µg/mL)	3–4 × normal
**Thrombocytopenia (platelets)**	100,000–150,000/mcL	<100,000/mcL
**Lymphocyte count**	1000–1500/mcL	—
**Lactate levels**	2 × normal	—
**PaO_2_/FiO_2_ ratio**	—	≤300 mmHg
**Respiratory rate**	—	>30 breaths per minute
**IL-6 (Interleukin-6)**	—	Elevated

**Table 3:** Pandemic waves in Romania.

Pandemic Wave	Time Period	Dominant Variant
**Wave 1**	January–June 2020	Wuhan-Hu-1 (NCBI RefSeq: NC_045512.2)
**Wave 2**	July–December 2020	Clade variant S: D614G
**Wave 3**	January–March 2021	Alpha (B.1.1.7)
**Wave 4**	September–November 2021	Delta (B.1.617.2)
**Wave 5**	January–March 2022	Omicron (B.1.1.529)
**Wave 6**	April–November 2022	Omicron subvariant BA.2
**Wave 7**	December 2022–March 2023	Omicron subvariants BQ.1.1 & XBB.1.5
**Wave 8**	June–August 2023	Omicron XBB.1.5
**Wave 9**	November 2023–April 2024	Omicron XBB.1.5
**Wave 10**	May–October 2024	JN.1 subvariant

**Table 4:** Patient characteristics.

Characteristics	Values
Age (mean ± SD)	67.26 ± 13.57
Gender (male)	55.03%
Urban (n=), %	63.08%
Obesity (n=), %	41.89%
**Blood count**	
WBC × 10^3^/µL (mean ± SD)	10,893.41 ± 11,593.79
Neutrophils × 10^3^/µL (mean ± SD)	9298.14 ± 10,869.27
Lymphocyte × 10^3^/µL (mean ± SD)	885.68 ± 393.62
Platelets × 10^3^/µL (mean ± SD)	220,993.24 ± 107,652.03
Creatinine mg/dL (mean ± SD)	1.04 ± 0.46
Total bilirubin mg/dL (mean ± SD)	0.72 ± 0.98
Direct bilirubin mg/dL (mean ± SD)	0.51 ± 0.43
GOT U/L (mean ± SD)	54.25 ± 40.74
GPT U/L (mean ± SD)	64.91 ± 52.93
GGT U/L (mean ± SD)	77.32 ± 47.51
FBG mg/dL (mean ± SD)	146.40 ± 63.81
NA mmol/L (mean ± SD)	135.40 ± 3.78
K mmol/L (mean ± SD)	4.08 ± 0.59
Total proteins g/dL (mean ± SD)	6.20 ± 0.69
Albumin/dL (mean ± SD)	3.55 ± 0.53
Procalcitonin ng/mL (mean ± SD)	0.99 ± 1.90
Cholesterol ng/mL (mean ± SD)	199.46 ± 50.28
LDL mg/dL (mean ± SD)	105.44 ± 31.79
HDL mg/dL (mean ± SD)	49.28 ± 18.65
Total lipids (mean ± SD)	678.18 ± 97.47
Triglycerides mg/dL(mean ± SD)	148.90 ± 55.44
Plasma calcium mg/dL (mean ± SD)	7.56 ± 1.94
LDH U/L (mean ± SD)	592.41 ± 270.02
Lactate mmol/L (mean ± SD)	21.39 ± 7.46
CRP mg/dL (mean ± SD)	10.09 ± 85.20
VSH mm/h (mean ± SD)	74.01 ± 35.61
Fibrinogen mg/dL (mean ± SD)	5.54 ± 1.53
Ferritin ng/mL (mean ± SD)	1114.47 ± 921.16
D-dimers ng/mL (mean ± SD)	4265.20 ± 3480.29
IL6 pg/mL (mean ± SD)	214.29 ± 705.68
APTT seconds (mean ± SD)	22.26 ± 3.41
INR (mean ± SD)	1.26 ± 1.14
**Comorbidities**	
Sepsis (n=), %	(24) 14.45%
Cancer (n=), %	(24) 14.45%
Hypertension (n=), %	(131) 78.9%
Pulmonary hypertension (n=), %	(45) 27.1%
COPD * (n=), %	(14) 8.43%
Asthma (n=), %	(10) 6.02%
Hematologic disease (n=), %	(4) 2.4%
Diabetes (n=), %	(51) 30.72%
CKD * (n=), %	(22) 13.25%
NYHA * 1	(29) 17.46%
NYHA 2	(50) 30.13%
NYHA 3	(35) 21.08%
NYHA 4	(1) 0.6%
Old myocardial infarction (n=), %	(1) 0.6%
Venous insufficiency (n=), %	(21) 12.65%
Atrial fibrillation (n=), %	(18) 10.84%
Stroke (n=), %	(11) 6.62%
Dementia (n=), %	(4) 2.4%
Cortical atrophy (n=), %	(4) 2.4%
**Treatment**	
Thrombolysis (actilyse)	(10) 6.02%
Nadroparin	(66) 37.95%
Sodium heparin	(28) 16.86%
Enoxaparin	(74) 44.57%
Remdesivir	(135) 81.32%
Favipiravir	(28) 16.86%
Tocilizumab	(18) 10.84%
Anakinra	(59) 35.54%
Dexamethasone	(98) 59.03%
Antiplatelet agent	(118) 71.08%
Anticoagulant therapy (n=), %	(61) 36.74%
**Others**	
PESI score (mean ± SD)	3.81 ± 1.08
Wells score (mean ± SD)	1.19 ± 1.52
Score IMPROVE-VTE (mean ± SD)	4.89 ± 1.66
Vaccinated (n=), %	(47) 28.31%
Days until onset (mean ± SD)	9.07 ± 4.56
Intubation (n=), %	(31) 18.67%
CPAP (n=), %	(31) 18.67%
Death (n=), %	(41) 24.69%

* COPD: chronic obstructive pulmonary disease; CKD: chronic kidney disease; NYHA: New York Heart Association.

**Table 5:** One-way ANOVA.

		Sum of Squares	df	Mean Square	F	Sig.
CRP	Between Groups	7811.196	1	7811.196	1.077	0.301
	Within Groups	1,066,401.118	147	7254.429		
	Total	1,074,212.313	148			
ESR	Between Groups	1902.380	1	1902.380	1.506	0.222
	Within Groups	185,731.593	147	1263.480		
	Total	187,633.973	148			
Fibrinogen	Between Groups	3.111	1	3.111	1.333	0.250
	Within Groups	343.029	147	2.334		
	Total	346.140	148			
Ferritin	Between Groups	1,368,309.742	1	1,368,309.742	1.619	0.205
	Within Groups	124,216,325.372	147	845,009.016		
	Total	125,584,635.114	148			
D-dimers	Between Groups	203,581.434	1	203,581.434	0.017	0.897
	Within Groups	1,792,433,497.542	147	12,193,425.153		
	Total	1,792,637,078.976	148			
IL6	Between Groups	129,920.055	1	129,920.055	0.260	0.611
	Within Groups	73,572,169.598	147	500,490.950		
	Total	73,702,089.653	148			

**Table 6:** Case processing summary.

		n=	%
Cases available in analysis	Event *	41	24.7
	Censored	122	73.5
	**Total**	**163**	**98.2**
Cases dropped	Cases with missing values	0	0.0
	Cases with negative time	0	0.0
	Censored cases before the earliest event in a stratum	3	1.8
	**Total**	**3**	**1.8**
**Total**		**166**	**100**

* Dependent variable: days until onset.

**Table 7:** Comparison of mean and median survival times by intubation status.

Means and Medians for Survival Time								
Intubation_Status	Mean ^a^				Median			
	Estimate	Std. Error	95% Confidence Interval		Estimate	Std. Error	95% Confidence Interval	
			Lower Bound	Upper Bound			Lower Bound	Upper Bound
0	8.696	0.398	7.915	9.477	7.000	0.414	6.188	7.812
1	8.484	0.800	6.915	10.053	8.000	0.687	6.654	9.346
Overall	8.657	0.356	7.959	9.354	7.000	0.358	6.299	7.701

^a^ Estimation is limited to the largest survival time if it is censored.

**Table 8:** Comparison of mean and median survival times by pandemic wave group.

Means and Medians for Survival Time								
Wave_Group	Mean ^a^				Median			
	Estimate	Std. Error	95% Confidence Interval		Estimate	Std. Error	95% Confidence Interval	
			Lower Bound	Upper Bound			Lower Bound	Upper Bound
1–5	9.477	0.418	8.659	10.296	9.000	0.691	7.646	10.354
6–10	7.088	0.614	5.885	8.291	6.000	0.594	4.836	7.164
Overall	8.657	0.356	7.959	9.354	7.000	0.358	6.299	7.701

^a^ Estimation is limited to the largest survival time if it is censored.

**Table 9:** Comparison of mean and SD across pandemic waves for Wells, Pessi, and IMPRUVE-VTE scores.

Wave		Wells		Pessi		IMPRUVE-VTE	
		Mean	SD	Mean	SD	Mean	SD
1	n = 2	0.75	0.75	2	1	3	1
2	n = 26	1.3	1.33	3.53	1.24	4.53	1.8
3	n = 33	0.8	1.02	3.48	1.07	4.39	1.45
4	n = 22	1.11	1.91	3.68	0.92	4.77	1.75
5	n = 26	1.09	1.26	4.07	0.87	4.88	1.28
6	n = 17	1.17	1.31	4.17	0.78	6	1.41
7	n = 15	2.53	2.08	4.25	1.08	5.75	1.78
8	n = 8	2.27	2.7	4.12	0.59	4.87	0.78
9	n = 9	1.18	0.78	4.88	0.31	5.66	1.15
10	n = 8	1.42	0.82	4.28	0.88	5.71	0.45

**Table 10:** Kendall’s tau-b correlation coefficient.

	Kendall’s tau-b	Significance (2-Tailed)	95% Confidence Intervals (2-Tailed) *	
			Lower	Upper
Wells_mean—Pessi_mean	0.619	0.051	−0.025	0.900
Wells_mean—IMPRUVE_VTE_mean	0.524	0.099	−0.165	0.869
Wells_mean—Wave	0.524	0.099	−0.165	0.869
Pessi_mean—IMPRUVE_VTE_mean	0.905	0.004	0.635	0.978
Pessi_mean—Wave	0.905	0.004	0.635	0.978
IMPRUVE_VTE_mean—Wave	0.810	0.011	0.361	0.954

* Estimation is based on Fisher’s r-to-z transformation.

**Table 11:** Clinical outcomes and lung involvement across COVID-19 pandemic waves.

Wave	Deaths		Intubation		Lung Involvement Imaging Features	
					Mean ± SD	
1	n = 1	0.6%	n = 1	0.6%	0.22	0.02
2	n = 7	4.21%	n = 3	1.8%	0.45	0.17
3	n = 8	4.81%	n = 8	4.81%	0.45	0.24
4	n = 6	3.61%	n = 2	1.2%	0.54	0.18
5	n = 6	3.61%	n = 4	2.4%	0.35	0.2
6	n = 6	3.61%	n = 5	3%	0.31	0.19
7	n = 2	1.2%	n = 4	2.4%	0.36	0.13
8	n = 0	0	n = 1	0.6%	0.24	0.12
9	n = 3	1.8%	n = 1	0.6%	0.33	0.17
10	n = 2	1.2%	n = 2	1.2%	0.31	0.31

**Table 12:** Pulmonary involvement across each wave.

		Pulmonary Involvement		
Wave	Total No.	0–20%	20–50%	>50%
1	n = 2	0	50% (1)	50% (1)
2	n = 26	19.2% (5)	38.4% (10)	42.3% (11)
3	n = 33	18.1% (6)	42.4% (14)	39.3% (13)
4	n = 22	4.5% (1)	36.3% (8)	59% (13)
5	n = 26	30.7% (8)	34.6% (9)	34.6% (9)
6	n = 17	47% (8)	29.4% (5)	23.5% (4)
7	n = 15	46.6% (7)	40% (6)	20% (3)
8	n = 8	40% (4)	37.5% (3)	12.5% (1)
9	n = 9	9 (100%)	0	0
10	n = 8	87.5% (7)	12.5% (1)	0

## Data Availability

The original contributions presented in the study are included in the article, further inquiries can be directed to the corresponding authors.
